# Awareness of Effective Communication Skills Among Paediatric Dentists in Turkey

**DOI:** 10.3290/j.ohpd.c_2680

**Published:** 2026-06-19

**Authors:** Murat Büyüksefil, Ülhak Çimen, Sera Şimşek Derelioğlu, Sait Sinan Atılgan, Feride Aktaş

**Affiliations:** a Murat Büyüksefil Paediatric Dentist, Department of Paediatric Dentistry, Atatürk University Faculty of Dentistry, Erzurum, Turkey. Conceptualisation, investigation, data curation, resources, writing – original draft, writing – review and editing, project administration, funding acquisition.; b Ülhak Çimen Associate Professor Dr, Department of Radio, Cinema and Television, Atatürk University Faculty of Communication, Erzurum, Turkey. Conceptualisation, methodology, survey validation, writing – original draft, funding acquisition, validation.; c Sera Şimşek Derelioğlu Paediatric Dentist, Professor Dr, Department of Pediatric Dentistry, Atatürk University Faculty of Dentistry, Erzurum, Turkey. Conceptualisation, investigation, data curation, resources, writing – original draft, writing – review and editing, supervision, project administration, funding acquisition.; d Sait Sinan Atılgan Associate Professor Dr, Department of Radio, Cinema and Television, Atatürk University Faculty of Communication, Erzurum, Turkey. Methodology, survey validation, formal analysis, funding acquisition, and validation.; e Feride Aktaş Paediatric Dentist, Professor Dr, Pediatric Dentist, Department of Pediatric Dentistry, Atatürk University Faculty of Dentistry, Erzurum, Turkey. Investigation, writing – review and editing.

**Keywords:** communication with children, communication skills, empathy, paediatric dentist, Turkey

## Abstract

**Purpose:**

Effective communication with paediatric patients is one of the cornerstones of paediatric dentistry. Strong communication skills play a critical role in reducing dental anxiety, enhancing patient satisfaction, and facilitating the overall treatment process. This study aimed to assess the verbal and nonverbal communication skills of paediatric dentists working across Turkey and to explore variations based on demographic variables.

**Methods and Materials:**

This descriptive cross-sectional study was conducted through an online questionnaire completed by 331 actively practising paediatric dentists in Turkey. The questionnaire included items structured on a 5-point Likert scale designed to evaluate communication competencies. Data were analysed using IBM SPSS Statistics 25.0. Descriptive statistics (frequency, percentage, mean) were used to summarise the participants’ responses, and comparative analyses were performed based on selected variables (eg, gender, years of professional experience, parenthood status). The Mann–Whitney U test was applied for comparisons between two groups when parametric test assumptions were not met. Furthermore, questionnaire items were grouped under specific sub-dimensions of communication and presented thematically. A significance level of *P* < 0.05 was adopted.

**Results:**

Overall, participants reported a high level of perceived self-efficacy regarding their communication skills. Communication scores of faculty members and private practitioners were significantly higher than those of research assistants (*P* < 0.001). A significant positive correlation was found between years of clinical experience and communication competencies (*P* < 0.01). Notably, younger clinicians scored lower in subdomains such as the use of ‘I-messages’ and democratic guidance.

**Conclusions:**

While the communication skills of paediatric dentists appear to be generally strong, younger clinicians may require further support in developing certain experience-based communication strategies. These findings highlight the need to integrate structured and practice-oriented communication training into both undergraduate and postgraduate dental education.

In paediatric dentistry, effective communication between the dentist and the child has a direct impact on treatment outcomes and the overall patient experience.^24^ Studies have shown that communication not only enhances the efficiency of the dentist and the child’s self-confidence but also significantly reduces dental anxiety and fear in children.^11^ Therefore, a patient-centred approach should be adopted; the dentist must use age-appropriate, simple, and comprehensible language, demonstrate empathy through nonverbal cues such as facial expressions and eye contact, and allocate sufficient time to the child.^5,11^ Ultimately, effective communication is a critical factor in increasing the success of dental treatments and improving patient satisfaction.^24^

Since paediatric patients differ from adults in terms of cognitive and emotional development, communication in paediatric dentistry requires specific strategies. Young children, in particular, are more prone to dental fear due to their immature coping mechanisms.^6,17^ For this reason, paediatric dentists often employ techniques such as ‘tell-show-do’ to explain each step of the procedure in advance, using age-appropriate analogies to provide reassurance.^19^ Furthermore, empathetic communication plays a key role; a pilot study demonstrated that empathetic interaction was significantly correlated with treatment success.^25^ Using language and behaviours appropriate to the child’s developmental level, avoiding fear-inducing expressions, and building a sense of trust can enhance the child’s compliance and cooperation during treatment.^21^

Empathy and trust directly influence a child’s cooperation in paediatric dental care. Supportive and understanding communication with the child has been shown to reduce behavioural anxiety responses.^28^ A dentist’s sensitive and empathetic demeanour significantly lowers the child’s anxiety during dental visits.^27^ Moreover, as the child’s trust in the dentist increases, their attitude towards treatment becomes more positive; in a trusting environment, children are more willing to accept proposed procedures and adhere better to recommendations.^10,13^ Therefore, establishing open, empathetic, and trust-based communication in paediatric dentistry contributes to patient engagement and promotes long-term positive oral health behaviours, ultimately improving clinical outcomes.^7,25^

In the international literature, communication skills are emphasised as an integral component of professional competence in dentistry.^20^ Research highlights the growing need for patient-centred communication training within dental education programmes. It is suggested that students develop communication skills through role-playing, video recordings, and direct patient interaction.^15,26^ In paediatric settings in particular, establishing a trusting relationship with both the child and the parent from the first visit is considered essential for successful communication.^14^ This approach requires the use of age-appropriate language, consistent demonstration of empathy, and the ongoing development of mutual trust.^11^

In Turkey, studies on communication skills within dental education remain limited. According to recent reviews, only about half of the approximately 77 dental faculties in the country offer courses on communication, most of which are elective.^5^ This represents a significant gap in both the instruction of communication skills and the awareness of paediatric dentists regarding this issue. Therefore, the present study aims to assess the awareness of paediatric dentists practising in Turkey regarding effective communication skills in paediatric dental care.

## METHODS AND MATERIALS

Ethical approval for this study was obtained from the Ethics Committee of Atatürk University Faculty of Dentistry (approval no: 2025/23). The study was conducted in accordance with the ethical principles of the Declaration of Helsinki and was reported following the Checklist for Reporting Results of Internet E-Surveys (CHERRIES) guidelines. The questionnaire was developed using Google Forms and was distributed exclusively to invited paediatric dentists. Informed consent was obtained online from all participants, who agreed to the scientific use of their data. All responses were collected anonymously and preserved in accordance with data confidentiality principles.

### Study Design

This study aimed to assess the awareness levels of paediatric dentists across Turkey regarding effective communication skills in paediatric dental practice. A quantitative research design was adopted using a descriptive survey model. The questionnaire was developed by a communication expert and two academic paediatric dentists (ÜÇ, MB, and SŞD) and was initially submitted to 15 field experts for content validation. Based on the expert feedback, the items were revised and finalised.

The pilot testing process consisted of two phases. In the first phase, a preliminary pilot was conducted with 25 paediatric dentists, and the items were revised based on their feedback regarding clarity and content. In the second phase, the revised version was administered to 40 additional paediatric dentists. The final version of the questionnaire was established after this two-stage pilot. Participants from the pilot phase were excluded from the main analysis to prevent potential bias.

### Population and Sample

Based on a population of approximately 1,200 paediatric dentists in Turkey, the required sample size was calculated with a 95% confidence level and a 5% margin of error. Assuming maximum variance (*P* = 0.5), the formula ‘*n = [Z* ^2^  *× p × (1 – p)] / e* ^2^’ was used. Applying finite population correction with a Z-score of 1.96, the minimum required sample size was determined to be 291 participants. Ultimately, 331 paediatric dentists who met the inclusion criteria and were actively practising across various regions in Turkey were included in the final analysis.

### Participants and Questionnaire Structure

The participants consisted of paediatric dentists who regularly treat paediatric patients and have varying levels of clinical experience in paediatric dental care. The questionnaire included demographic variables such as gender, age group, academic title, and years of professional experience. In the final stage of the study, a total of 331 paediatric dentists who were actively practising across various provinces of Turkey and met the inclusion criteria were included in the sample.

The questionnaire consisted of 29 items, each rated on a five-point Likert scale. The items were not adapted from any standardised instrument but were originally developed based on a comprehensive literature review and expert opinions. The survey was self-administered and structured to assess paediatric dentists’ awareness of various components of effective communication with children. These components included empathy, nonverbal communication skills (eg, body language and eye contact), use of ‘I-statements’, awareness of verbal and non-verbal messaging, sensitivity to cultural differences, and attention to digital distractions.

### Thematic Grouping and Internal Consistency

Survey items were grouped thematically and internal consistency was assessed using Cronbach’s alpha. Items were categorised into four primary themes:

**Verbal communication skills:** Competencies related to language and tone of voice (eg, avoiding jargon, using positive language, adjusting tone appropriately) – items 9, 17, 18, 19, 20, 21.
**Body language (non-verbal communication):** Use of eye contact, body posture, touch, gestures, and facial expressions – items 11, 12, 13, 14, 15.
**Empathy and active listening:** Understanding and valuing the child’s emotions, providing feedback, and demonstrating a positive attitude – items 3, 4, 5, 6, 7, 10, 16, 22, 23, 24, 25.
**Sensitivity to cultural and individual differences:** Ability to adapt communication to the child’s age, developmental stage, personality, and cultural background – items 1, 2, 8.

The average scores of each thematic group were calculated. Additionally, a subscale titled ‘Motivation and Behavioural Outcomes’ (items 26, 27, 28, 29) was analysed separately to assess the perceived impact of effective communication on treatment motivation and behavioural change.

Cronbach’s alpha values for internal consistency were as follows:

Verbal communication: α = 0.60 (6 items)
Body language: α = 0.76 (5 items)
Empathy/active listening: α = 0.80 (11 items)
Cultural/individual sensitivity: α = 0.55 (3 items)
Motivation outcomes: α = 0.77 (4 items)

### Data Collection Process

Data were collected during the spring term of 2025 on a voluntary basis. Participants were informed about the purpose and scope of the study, and data collection was carried out in accordance with ethical standards following informed consent.

### Statistical Analysis

Data analysis was conducted using IBM SPSS Statistics version 25.0. Descriptive statistics (frequency, percentage, mean) were used to summarise the distribution of responses. The Mann–Whitney U test was applied for comparisons between two independent groups where parametric assumptions were not met. Comparative analyses were performed based on variables such as gender, years of experience, and parental status. The questionnaire items were presented under the thematic subdimensions described above.

## RESULTS

The demographic information regarding gender, age distribution, parental status, academic title, and years of professional experience is presented in Table 1. The majority of the participants were female, and a substantial proportion of the sample was within the 25–30 age group.

**Table 1:** Demographic profile of the study sample

Variable	Category	n	%
Gender	Female	297	89.7
	Male	34	10.3
Age	25–30	219	66.2
	30–35	80	24.2
	35–40	17	5.1
	40–45	10	3.0
	45–50	2	0.6
	≥ 50 years	3	0.9
Parental status	No	265	80.1
	Yes	66	19.9
Academic title	Research assistant (DDS)	149	45.0
	Specialist dentist (DDS, PhD)	148	44.8
	Assistant professor (DDS, PhD)	20	6.0
	Associate professor (DDS, PhD)	8	2.4
	Professor (DDS, PhD)	6	1.8
Years of professional experience	0–4 years	207	56.3
	5–9 years	82	22.3
	10–14 years	23	6.3
	15 years and more	18	4.9


### Descriptive Statistics

A total of 331 paediatric dentists actively working in various provinces of Turkey participated in the study. The questionnaire consisted of 29 items, each rated using a five-point Likert scale. The items were developed based on expert opinions and were not adapted from any standardised scale. The aim of the questionnaire was to assess paediatric dentists’ approaches to various aspects of communication with children, including verbal and nonverbal communication skills, empathy, use of ‘I-statements’, sensitivity to cultural differences, and attentiveness to digital/technological distractions.

According to the descriptive statistics of the questionnaire, the mean scores for many items were found to be above 4.0. For example, the item ‘I prefer to use child-friendly language during communication with children’ had a mean score of 4.47, with 99% of responses marked as either ‘Agree’ or ‘Strongly Agree’. The item ‘I behave in an optimistic, kind, affectionate, and cheerful manner towards children’ had a mean score of 4.51. Similarly, the statement ‘I apply communication methods appropriate to different stages of childhood’ had a mean of 4.47 and was one of the highest-rated items, with 99% of participants providing positive responses. The item ‘A child’s clothing, ethnicity, or accent does not affect my behaviour’ received a mean score of 4.42, with 93% of responses marked as ‘Strongly Agree’. The statement ‘I listen to the child carefully and attentively’ had a mean score of 4.46, with 96% of participants responding positively. Detailed data on the mean scores, standard deviations, and response distribution percentages for each item are presented in Table 2, Table 3, and Table 4.

**Table 2:** Descriptive statistics for the items of the communication skills questionnaire (questions 1–10)

Question	Mean	Standard deviation	Strongly disagree (%)	Disagree (%)	Undecided (%)	Agree (%)	Strongly agree (%)
Q01	4.47	0.52	0.0	0.0	1.2	50.8	48.0
Q02	4.37	0.58	0.0	0.3	3.3	55.0	41.4
Q03	4.31	0.63	0.0	0.6	6.3	55.3	37.8
Q04	4.35	0.61	0.0	0.6	4.8	54.7	39.9
Q05	4.27	0.59	0.0	0.0	9.7	56.8	33.5
Q06	4.38	0.63	0.0	0.3	5.4	50.8	43.5
Q07	4.30	0.60	0.0	0.3	4.8	57.1	37.8
Q08	4.42	0.80	1.2	1.2	4.5	33.8	59.2
Q09	4.47	0.56	0.0	0.0	3.0	52.0	45.0
Q10	3.98	1.02	3.6	10.6	19.9	34.4	31.4


**Table 3:** Descriptive statistics for the items of the communication skills questionnaire (questions 11–20)

Question	Mean	Standard deviation	Strongly disagree (%)	Disagree (%)	Undecided (%)	Agree (%)	Strongly agree (%)
Q11	4.34	0.62	0.0	0.3	4.2	55.3	40.2
Q12	4.40	0.60	0.0	0.0	3.0	53.2	43.8
Q13	4.30	0.69	0.0	0.6	7.3	49.5	42.6
Q14	4.38	0.64	0.0	0.3	4.5	52.0	43.2
Q15	4.29	0.64	0.0	0.3	6.0	56.5	37.2
Q16	4.51	0.58	0.0	0.0	1.8	44.7	53.5
Q17	4.29	0.69	0.3	0.3	6.3	53.5	39.6
Q18	3.98	0.85	0.9	5.4	18.7	46.5	28.4
Q19	3.10	1.03	5.4	22.4	39.3	23.0	10.0
Q20	4.38	0.64	0.3	1.5	4.8	50.5	42.9


**Table 4:** Descriptive statistics for the items of the communication skills questionnaire (questions 21–29)

Question	Mean	Standard deviation	Strongly disagree (%)	Disagree (%)	Undecided (%)	Agree (%)	Strongly agree (%)
Q21	4.24	0.66	0.3	1.5	8.5	52.3	37.5
Q22	4.40	0.54	0.0	0.0	4.8	55.6	39.6
Q23	4.46	0.55	0.0	0.0	3.6	53.2	43.2
Q24	4.15	0.66	0.3	1.8	11.2	54.1	32.6
Q25	3.66	1.13	4.8	14.2	28.7	40.5	11.8
Q26	4.43	0.55	0.0	0.0	3.0	57.7	39.3
Q27	4.44	0.53	0.0	0.0	2.4	56.8	40.8
Q28	4.14	0.71	0.0	0.6	12.7	57.1	29.6
Q29	4.14	0.74	0.0	2.4	14.2	50.5	32.9


On the other hand, relatively lower mean scores were obtained for some questionnaire items. For instance, the item ‘I use I-messages when communicating with children’ had a mean score of 3.10. An analysis of the response distribution revealed that 33% of participants selected ‘Strongly Agree’, 27% chose ‘Disagree’, and 39% were ‘Undecided’. Similarly, for the item ‘I adopt a democratic attitude’, the mean score was 3.66, with 19% of responses marked as ‘Disagree’, 52% as ‘Agree’, and 29% as ‘Undecided’.

According to the internal consistency analysis, the overall Cronbach’s alpha value for the scale was calculated as 0.92. When the subscales were examined, the internal consistency for the nonverbal communication (body language) and empathy subscales was found to be at the level of 0.70. The Cronbach’s alpha value was approximately 0.60 for the verbal communication subscale and 0.55 for the cultural and individual differences subscale.

### Communication Scores According to Demographic Variables

According to the results of the independent samples t-test conducted based on gender, no statistically significant difference was found in the communication skills scores between female (n = 297) and male (n = 34) paediatric dentists (*P* > 0.05). The mean total communication score was 4.22 for females and 4.26 for males.

The one-way ANOVA conducted for age groups revealed significant differences in communication scores among the 25–30 years (n = 219), 30–35 years (n = 80), and 35 years and older (n = 32) groups (*P* < 0.001). The highest mean score was observed in the 35 years and older group (4.50), while the lowest was in the 25–30 years’ group (4.18). According to post-hoc analyses, the 35+ age group had significantly higher scores compared to the other two groups (*P* < 0.01).

The analysis based on academic title also revealed significant differences (*P* < 0.001). Research assistants (n = 149) reported a mean score of 4.08, private practice paediatric dentists (n = 148) 4.33, and faculty members (n = 34) 4.35. Post-hoc tests indicated statistically significant differences between research assistants and the other two groups (*P *< 0.001), while no significant difference was found between private practitioners and faculty members.

The ANOVA conducted based on years of experience working with paediatric patients also showed statistically significant differences among the groups: 0–1 year (n = 62), 2–5 years (n = 184), 6–10 years (n = 57), and 11 years and above (n = 28) (*P* < 0.001). The highest mean score was observed in the 11+ years group (4.59), while the lowest was in the 0–1-year group (4.03). According to post-hoc analyses, the 11+ years group scored significantly higher than the 0–1 year and 6–10 year groups (*P* < 0.01). The scores for the 2–5-year and 6–10-year groups were similar (~4.22–4.25).

## DISCUSSION

Effective communication skills are a fundamental component of paediatric dental practice, as they directly influence child cooperation, treatment success, and the overall dental experience. In paediatric dentistry, communication is not limited to verbal interaction but also includes non-verbal communication, behaviour guidance, and the establishment of trust between the dentist, the child, and the parents. Strong communication skills enable clinicians to reduce dental anxiety, improve cooperation, and facilitate the delivery of dental treatment under local anaesthesia rather than general anaesthesia.^2,4,12^

This study aims to evaluate the verbal and nonverbal communication skills used by 331 paediatric dentists actively practising across Turkey in their interactions with child patients. The findings indicate that paediatric dentists have a high level of self-efficacy regarding their communication skills and that they adopt child-friendly approaches during communication processes.

In recent years, there has been an increasing emphasis on reducing unnecessary general anaesthesia in paediatric dental patients due to the potential medical risks, high cost, and psychological impact associated with hospital-based treatment. Effective communication and behaviour guidance strategies play a critical role in managing children in the dental chair and may help prevent the need for pharmacological management techniques. Therefore, improving communication skills among dental students and young dentists may contribute to a reduction in general anaesthesia cases by increasing the success of chairside behaviour management.^2,3^

The survey results particularly highlight the high mean scores in both verbal and nonverbal communication domains. For instance, the item ‘I prefer to use child-oriented language during communication’ received 99% agreement, with a mean score of 4.47. Similarly, the statement ‘I behave optimistically, kindly, affectionately, and with a smile toward children’ yielded one of the highest mean scores, at 4.51. These findings demonstrate that paediatric dentists adopt an empathetic, supportive, and warm communication style with their young patients. Likewise, the item ‘I use communication methods appropriate to different stages of childhood’ showed a 99% agreement rate and a mean score of 4.47, indicating widespread use of age-appropriate communication strategies. These data align with the existing literature emphasising the importance of child-centred dental practices.^2,9^

Participants also showed high levels of sensitivity to cultural and individual differences. The item ‘A child’s clothing, ethnic identity, or accent does not alter my behaviour’ received a 93% positive response rate and a mean score of 4.42, suggesting that paediatric dentists tend to adopt a nonjudgmental and egalitarian approach. Furthermore, the statement ‘I listen to the child carefully and attentively’ had a 96% agreement rate and a mean score of 4.46, underscoring the strong presence of active listening in communication practices. Indeed, Sarnat et al^21^ analysed the communication styles of paediatric dental interns treating patients aged 3–12 and found that empathetic approaches had the strongest correlation with children’s cooperation levels, treatment success rates, and post-treatment emotional states, surpassing other verbal style categories such as ‘permissive’ and ‘personal’ approaches. In the same vein, the American Academy of Paediatric Dentistry’s 2025 guideline, ‘Behavior Guidance for the Paediatric Dental Patient’, emphasises that active listening, empathetic verbal and nonverbal communication, and sensitivity to cultural and individual differences form a foundation of trust. These patient- and family-centred strategies directly enhance child and parent engagement in treatment, reduce anxiety, facilitate cooperation, and improve treatment outcomes.^2^

On the other hand, areas in need of improvement were also identified. The item ‘I use I-messages when communicating with children’ had a mean score of 3.10, with 27% of participants disagreeing and 39% remaining undecided. This finding suggests that the use of ‘I-messages’ has not been sufficiently integrated into clinical practice and highlights the need for increased awareness and training in this area. Additionally, the statement ‘I adopt a democratic attitude’ had a mean score of 3.66, with 19% of respondents indicating disagreement, suggesting that participatory approaches in communication with children have not been fully embraced by all practitioners.

Internal consistency analyses indicated that the scale used in this study was highly reliable, with an overall Cronbach’s α of 0.92. When analysed by subscales, the domains of body language and empathy showed acceptable reliability (α = 0.70), while the verbal communication (α = 0.60) and cultural sensitivity (α = 0.55) subscales demonstrated moderate internal consistency. These findings suggest that the subscales of verbal communication and cultural sensitivity may require further psychometric strengthening.

Analyses based on demographic variables revealed that communication skills significantly increased with age and professional experience. For example, the mean communication score among paediatric dentists aged 35 and older was 4.50, compared to 4.18 in the 25–30 age group (*P* < 0.001). Similarly, participants with more than 11 years of experience reported a mean score of 4.59, while those who had recently started their careers (0–1 year of experience) had a mean score of 4.03 (*P* < 0.001). These findings indicate that communication skills improve over time and that clinical experience plays a crucial role in this development. Indeed, this trend is consistent with the literature suggesting that communication is a skill acquired progressively, enhanced through practice, and significantly reinforced by clinical experience.^20,23^

These findings reveal that faculty members (mean = 4.35) and clinical practitioners (mean = 4.33) reported significantly higher communication skills scores compared to research assistants (mean = 4.08) (*P* < 0.001). This suggests that academic rank, often associated with professional maturity, is reflected in communication competencies, and this pattern aligns with structural models described in the literature.^16,22^

The vast majority of paediatric dentists reported using key communication skills such as active listening, empathy, and providing positive motivation effectively. These abilities play a crucial role in positively influencing children’s treatment experiences. The observed positive relationship between clinical experience and communication scores supports the notion that growing professional confidence over time enhances communication abilities. Existing literature also suggests that experienced clinicians demonstrate more effective communication in challenging situations, such as managing anxious patients.^8^

The observed shortcomings in the use of ‘I-messages’ among younger paediatric dentists are noteworthy. The literature emphasises that ‘you-messages’, which attribute negative behaviours to a child’s personality, can be more confrontational and provoke resistance. In contrast, ‘I-messages’ are more effective in guiding behaviour and fostering empathetic communication.^18^ This highlights the need to teach communication skills through practical, experience-based training, particularly during the early stages of a dental career.

High scores in the cultural sensitivity dimension are also noteworthy. Paediatric dentists were observed to adopt a sensitive and egalitarian attitude toward patients’ ethnic backgrounds, language, and appearance. This suggests that a patient-centred approach and cultural competence are well-integrated into professional dental practice. However, the relatively low internal consistency of this subscale raises the possibility that combining cultural awareness and individual differences within a single dimension may present conceptual challenges. Future studies are recommended to explore this construct using a separate, dedicated scale.

Similarly, the relatively low internal consistency of the verbal communication subscale (α ≈ 0.60) compared to other sub-dimensions may be attributed to the structural diversity of its items. In particular, the low score for the ‘I-messages’ item appears to have negatively affected the overall consistency. These findings suggest that the verbal communication domain may need to be evaluated in terms of its distinct subcomponents.

The findings of this study indicate that paediatric dentists practising in Turkey demonstrate a high level of perceived competence in key paediatric communication strategies such as empathy, active listening, and effective verbal and nonverbal communication. However, the relatively lower scores among younger paediatric dentists in domains such as the use of ‘I-messages’, democratic guidance, and constructive feedback clearly highlight the need for these areas to be addressed early and through applied methods in communication training. The data also show that communication scores increase significantly with clinical experience, supporting the view that communication skills are learnable over time and are reinforced through practice.

Behaviour guidance techniques, as recommended by the American Academy of Paediatric Dentistry (AAPD), are an essential part of paediatric dental education. Techniques such as tell-show-do, positive reinforcement, distraction, non-verbal communication, voice control, and parental involvement are widely accepted as fundamental non-pharmacological behaviour management strategies. Dental education programmes should therefore place greater emphasis on communication training and behaviour guidance techniques to prepare future dentists for the clinical management of paediatric patients.^1,2^

The literature also emphasises that experienced clinicians are more effective in communicating with anxious, uncooperative, or resistant paediatric patients, and that this skill has a significant impact on establishing trust and achieving treatment success.^2,21^ Additionally, there is empirical evidence from social science-based research showing strong correlations between communication skills and lower dental anxiety, higher patient satisfaction, and better adherence to treatment.^11,20^

This study has several limitations. First, paediatric dentists’ communication skills were assessed solely through self-reported questionnaire data. Self-assessment may introduce social desirability bias and may not fully reflect actual behaviour in clinical settings. Furthermore, the absence of complementary methods, such as observational assessments or patient-reported outcomes, limits the extent to which these findings can be generalised to real-world practice. Another limitation is the voluntary nature of participation, which may have resulted in selection bias. Paediatric dentists who place greater emphasis on communication or feel more confident in this area may have been more inclined to participate in the study.

Despite these limitations, the findings offer important insights into paediatric dentists’ awareness and perceived competence in communicating effectively with children. In light of this, it is recommended that communication skills training be systematically and practically integrated into paediatric dentistry residency curricula at an early stage. Such training would serve as a strategic approach to strengthen the ability of young clinicians to engage in child-centred, empathetic, and interactive communication.

## CONCLUSION

This study showed that paediatric dentists generally perceive themselves as competent in communication with child patients; however, communication skills appear to improve with professional experience, highlighting the need for structured training, particularly for younger dentists. Effective communication and behaviour guidance skills play an important role in improving child cooperation and may help reduce the need for general anaesthesia. Therefore, communication and behaviour guidance training should be considered an essential component of paediatric dental education.

### Acknowledgements

#### Funding

None.

#### Data availability

All of the data produced or examined in the course of this research are presented within the content of this published article.

#### Ethics approval and consent to participate

Ethical approval for this study was granted by the Ethics Committee of Atatürk University Faculty of Dentistry (approval no: 2025/23). The study adhered to the principles of the Declaration of Helsinki and followed the CHERRIES guidelines.

#### Consent for publication

Not applicable.

#### Informed consent

Participation was voluntary, and informed consent was obtained online. All data were collected anonymously and handled in accordance with data confidentiality standards.

#### Competing interests

The authors declare no competing interests.
